# Hyperprogression after Anti-Programmed Death-1 Therapy in a Case of Sigmoid Colon Cancer with Lynch Syndrome

**DOI:** 10.70352/scrj.cr.25-0634

**Published:** 2026-04-09

**Authors:** Ryota Miura, Gaku Ohira, Michihiro Maruyama, Toru Tochigi, Tetsuro Maruyama, Koichiro Okada, Atsushi Hirata, Hisahiro Matsubara

**Affiliations:** Department of Frontier Surgery, Chiba University Graduate School of Medicine, Chiba, Chiba, Japan

**Keywords:** lynch syndrome, immune checkpoint inhibitor, microsatellite instability, hyperprogressive disease, pembrolizumab

## Abstract

**INTRODUCTION:**

Lynch syndrome is a hereditary condition caused by germline defects in mismatch repair (MMR) genes, resulting in microsatellite instability-high (MSI-H) tumors. Immune checkpoint inhibitors (ICIs) have demonstrated efficacy in the treatment of MSI-H advanced cancers. However, hyperprogressive disease (HPD) following ICI therapy is a rare and paradoxical phenomenon. We report a case of advanced sigmoid colon cancer associated with Lynch syndrome that developed HPD after pembrolizumab treatment.

**CASE PRESENTATION:**

A 51-year-old woman with advanced sigmoid colon cancer and a history of endometrial cancer presented with severe colonic stenosis, peritoneal dissemination, and suspected liver metastases, necessitating emergency colostomy. Immunohistochemistry revealed loss of MLH1 and PMS2 expression, consistent with suspected Lynch syndrome. Targeted molecular testing identified a *KRAS* exon 2 mutation (c.38G>A; p.G13D), with wild-type *NRAS* (exons 2–4) and *BRAF* (exon 15), and MSI-H status. Pembrolizumab was initiated, but within 3 days, rapid disease progression occurred, including abscess formation, enlargement of liver metastases, and lymphadenopathy, accompanied by clinical deterioration. Pembrolizumab was discontinued, and FOLFOXIRI plus bevacizumab was introduced, resulting in clinical stabilization. Subsequent germline testing confirmed Lynch syndrome with a pathogenic *MLH1* variant. At 24 months of chemotherapy, the patient remains alive with a sustained partial response.

**CONCLUSIONS:**

This case underscores the importance of early response evaluation in ICI-treated MSI-H tumors. Rapid disease progression requires prompt differentiation between HPD and pseudoprogression, emphasizing the necessity of timely therapeutic modification.

## Abbreviations

CA19-9
: carbohydrate antigen 19-9
CEA
: carcinoembryonic antigen
CRC
: colorectal cancer
ECOG PS
: Eastern Cooperative Oncology Group performance status
FDG
: fluorodeoxyglucose
HPD
: hyperprogressive disease
ICI
: immune checkpoint inhibitor
MMR
: mismatch repair
MSI-H
: microsatellite instability-high
PR
: partial response
SUVmax
: standardized uptake value maximum

## INTRODUCTION

Colorectal cancer (CRC) is the third most common cancer worldwide and remains a major cause of cancer-related mortality despite advancements in systemic therapies and surgical interventions.^1)^ A subset of CRC cases is attributable to Lynch syndrome, an autosomal dominant hereditary cancer syndrome caused by germline mutations in DNA mismatch repair (MMR) genes, leading to microsatellite instability-high (MSI-H) tumors.^2,3)^ Lynch syndrome accounts for approximately 3% of all CRC cases and is characterized by an elevated lifetime risk of colorectal and endometrial cancers, among others.^2,3)^

Immune checkpoint inhibitors (ICIs), such as pembrolizumab and nivolumab, have revolutionized the treatment of MSI-H or MMR-deficient (dMMR) CRC by targeting the programmed death-1 (PD-1) or programmed death ligand-1 (PD-L1) pathways, thereby restoring antitumor immune responses.^4,5)^ The KEYNOTE-177 trial established pembrolizumab as first-line therapy for MSI-H/dMMR metastatic CRC, demonstrating superior progression-free survival (16.5 vs. 8.2 months; hazard ratio [HR] = 0.60, P <0.0002) compared with chemotherapy.^4)^

Despite these promising results, some patients experience atypical treatment responses, including hyperprogressive disease (HPD), defined as accelerated tumor growth after ICI therapy.^6)^ Distinguishing HPD from pseudoprogression (PsP)—a transient tumor enlargement due to immune cell infiltration that later regresses with continued therapy—remains a significant clinical challenge.^7)^ Here, we describe a rare case of HPD in a patient with Lynch syndrome–associated sigmoid colon cancer following pembrolizumab initiation, highlighting the diagnostic and therapeutic challenges of differentiating HPD from PsP.

## CASE PRESENTATION

**Fig. 1:**
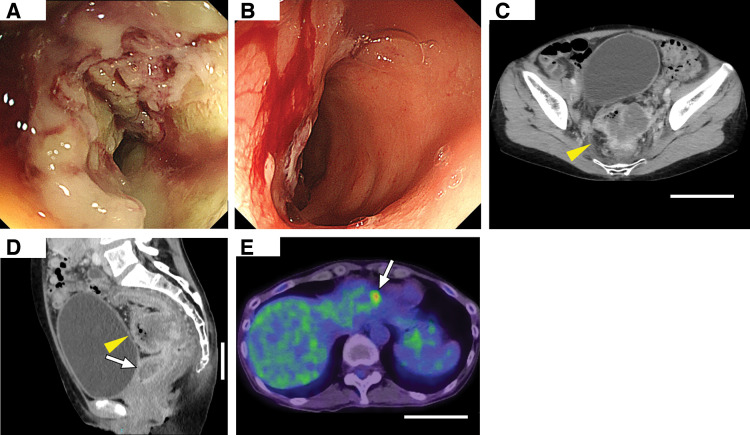
Endoscopic and radiologic findings at initial presentation (scale bar, 10 cm). (**A**) Lower gastrointestinal endoscopy revealed a type 1 tumor in the sigmoid colon. (**B**) Lower gastrointestinal endoscopy also demonstrated erosion in the rectum, suggesting possible invasion due to peritoneal dissemination. (**C**) Axial contrast-enhanced CT revealed the primary tumor in the sigmoid colon, measuring 62 × 44 mm. (**D**) Sagittal contrast-enhanced CT showed peritoneal dissemination in the Douglas pouch with suspected invasion into the rectum. (**E**) PET-CT showed a focal area of intense FDG uptake (SUVmax 5.7) in segment 3 (S3) of the liver, suggesting liver metastasis. FDG, fluorodeoxyglucose; SUVmax, standardized uptake value maximum

A 51-year-old woman presented with abdominal pain and constipation. At a previous hospital, colonoscopy revealed a type 1 tumor in the sigmoid colon (**Fig. 1A**), rectal erosion (**Fig. 1B**), and purulent vaginal discharge. She was referred to our department for further management.

Her history included endometrial cancer diagnosed 8 years earlier, treated with total hysterectomy and pelvic lymph node dissection at our institution. Family history was notable for multiple malignancies: uterine, colorectal, and bile duct cancers in her mother, and multiple myeloma and CRC in her brother. A pedigree analysis confirmed Lynch syndrome–associated tumors in three individuals across two generations.

Given the personal and family history suggestive of Lynch syndrome and the observed loss of MLH1/PMS2 by immunohistochemistry, genetic counseling was provided in our genetics outpatient clinic to the patient and her spouse. Pretest counseling included a review of the family history; an explanation of the autosomal-dominant inheritance pattern, the Lynch syndrome-associated cancer spectrum, and options for cancer prevention and surveillance; and a discussion of the clinical and familial implications of germline testing. Written informed consent was obtained, and germline testing was performed using a 29-gene hereditary cancer panel that included MMR genes. Posttest counseling was subsequently conducted to disclose the results and to discuss cascade testing and recommended surveillance for at-risk relatives.

On admission, laboratory tests showed normal carcinoembryonic antigen (CEA) (1.0 U/mL) and carbohydrate antigen 19-9 (CA19-9, 7.2 U/mL). Contrast-enhanced CT demonstrated a sigmoid tumor with severe stenosis (**Fig. 1C**), peritoneal seeding in the Douglas pouch with rectal invasion (**Fig. 1D**), and suspected liver metastases. PET-CT revealed intense fluorodeoxyglucose (FDG) uptake in the primary tumor and peritoneal deposits (standardized uptake value maximum [SUVmax] 19.23). Additional FDG uptake was noted in the lateral liver segment beneath the diaphragm (SUVmax 5.78) (**Fig. 1E**), supporting the diagnosis of liver metastasis.

**Fig. 2:**
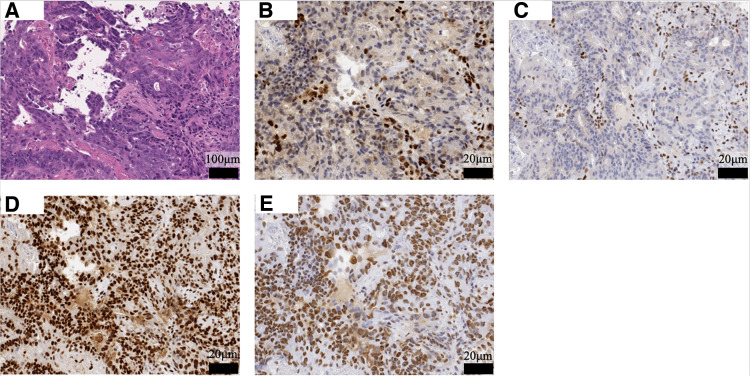
Histopathological examinations and immunohistochemical analysis (scale bars: 100 μm in A; 20 μm in **B**–**E**). (**A**) Hematoxylin and eosin staining revealed fused glandular structures with cribriform architecture, consistent with moderately differentiated adenocarcinoma (magnification: ×20). (**B**) Loss of MLH1 protein expression was detected in the tumor cells (magnification: ×100). (**C**) Loss of PMS2 protein expression was detected in the tumor cells (magnification: ×100). (**D**) Expression of MSH2 protein was preserved in the tumor cells (magnification: ×100). (**E**) Expression of MSH6 protein was preserved in the tumor cells (magnification: ×100).

Because of obstructive symptoms, an emergency transverse colostomy was performed. Histopathology of the biopsy specimen revealed adenocarcinoma (tub2 >por). Immunohistochemistry showed loss of MLH1 and PMS2 expression with preserved MSH2 and MSH6 (**Fig. 2**). Retrospective immunostaining of the prior endometrial tumor revealed identical findings. Targeted molecular testing identified a *KRAS* exon 2 mutation (c.38G>A; p.G13D), with wild-type *NRAS* (exons 2–4) and *BRAF* (exon 15), and MSI-H status.

**Fig. 3:**
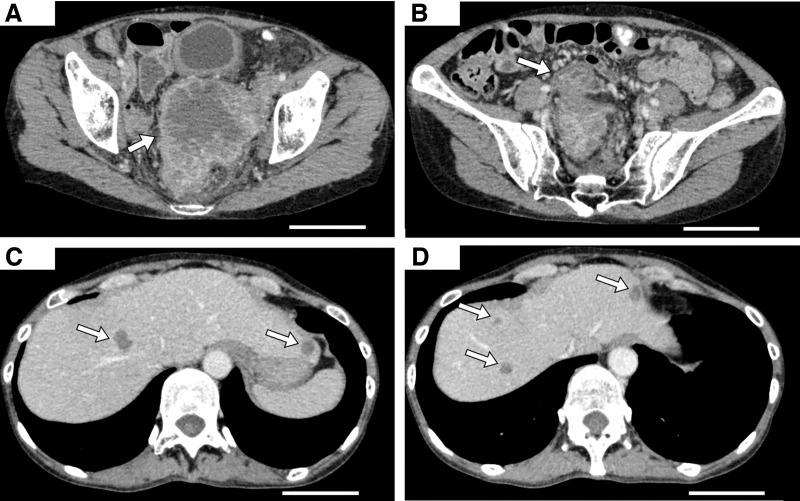
CT findings on day 7 after pembrolizumab (scale bar, 10 cm). (**A**) Rapid enlargement of the primary tumor (103 × 81 mm) with abscess formation was observed; the arrow indicates the enlarged primary tumor. (**B**) Newly developed lateral lymphadenopathy was noted; the arrow indicates the lateral lymph node. (**C**, **D**) Multiple new liver metastases were identified; the arrows indicate the newly developed liver metastases.

The patient was diagnosed with unresectable advanced sigmoid colon cancer (cT4a, cN1, cM1c [P2, H1], Stage IVc) with suspected Lynch syndrome. Pembrolizumab (200 mg every 3 weeks) was initiated as first-line therapy. Three days after the first dose, she presented with persistent abdominal pain and fever (38.6°C). Physical examination revealed tachycardia (107 bpm) and lower abdominal tenderness. Laboratory findings showed leukocytosis (12000/μL), elevated C-reactive protein (7.66 mg/dL), and lactate dehydrogenase (681 IU/L). Eastern Cooperative Oncology Group performance status (ECOG PS) worsened rapidly from 0 to 2. CT demonstrated rapid enlargement of the primary tumor with abscess formation (**Fig. 3A** and **3B**), progression of liver metastases (**Fig. 3C** and **3D**), and new lymphadenopathy, consistent with HPD.

**Fig. 4:**
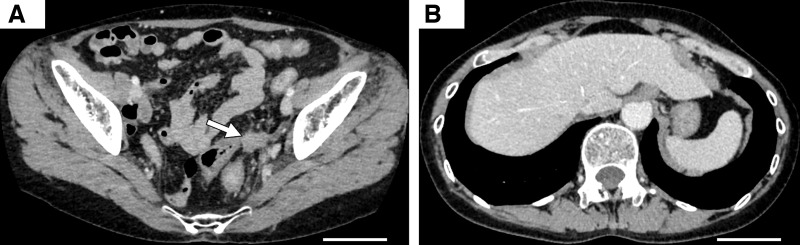
CT after four courses of FOLFOXIRI + bevacizumab (scale bar, 10 cm). (**A**) Significant reduction of the primary tumor, measuring 22 × 18 mm, with scar formation was observed (arrow). (**B**) Marked regression of liver metastases was noted.

On day 14 after pembrolizumab, therapy was switched to FOLFOXIRI plus bevacizumab due to rapid progression and clinical decline. The patient’s condition improved markedly, and she was discharged 11 days later. Subsequent germline testing identified a heterozygous *MLH1* splice-site variant (NM_000249.3:c.381-1G>C), classified as pathogenic. Aberrant splicing was confirmed by messenger RNA analysis from peripheral blood leukocytes, with a predicted frameshift (p.Arg127SerfsTer13), thereby confirming Lynch syndrome. At 3 months, follow-up imaging showed marked tumor regression, assessed as partial response (PR). She has remained on chemotherapy, and at 24 months, continues treatment with a sustained PR and no new metastases (**Fig. 4A** and **4B**).

## DISCUSSION

HPD is a severe and paradoxical clinical phenomenon defined by rapid tumor growth, shortened time to treatment failure, and abrupt deterioration in PS following ICI therapy. Ferrara et al. reported an incidence of HPD in approximately 13.8% of ICI-treated patients, with markedly poorer outcomes and a median overall survival of only 3.4 months compared with 6.2 months in patients without HPD.^6)^ Similarly, Champiat et al. described HPD as a >50% increase in tumor growth rate, occurring most often in non-small cell lung and gastric cancers, and emphasized the importance of close clinical monitoring during ICI therapy.^8)^

Lynch syndrome–associated tumors, typically characterized by MSI-H and dMMR, are generally expected to respond favorably to ICIs.^4,5)^ Both pembrolizumab and nivolumab have shown robust efficacy in MSI-H CRC, with significantly improved progression-free survival (PFS) compared to conventional chemotherapy. The KEYNOTE-177 trial established pembrolizumab as a preferred first-line therapy in this setting, demonstrating a median PFS of 16.5 months versus 8.2 months with chemotherapy (HR = 0.60, P <0.0002).^4)^ The favorable immunogenicity of MSI-H tumors, associated with high tumor mutational burden and abundant tumor-infiltrating lymphocytes, likely underlies this sensitivity to ICIs.^5,9)^ Nonetheless, a minority of MSI-H or Lynch syndrome–associated tumors paradoxically exhibit resistance or even HPD. The present case illustrates such a rare occurrence, with rapid and dramatic disease acceleration soon after pembrolizumab initiation.

A clinically similar but biologically distinct phenomenon is PsP, characterized by transient tumor enlargement due to immune cell infiltration followed by regression with continued therapy.^10,11)^ PsP occurs in only 5%–10% of patients and is usually associated with favorable outcomes.^10)^ Distinguishing PsP from HPD remains a major clinical challenge, as both conditions initially appear as radiologic disease progression, potentially delaying appropriate treatment modifications. Histopathological features of PsP, including dense immune infiltration and tumor necrosis, have been described.^11)^ In contrast, HPD has been linked to viable proliferating tumor cells, infiltration by M2-like tumor-associated macrophages expressing CD163, CD33, and PD-L1, and, in some cases, pathological transformation or altered PD-L1 expression.^12)^ In our patient, no biopsy was performed during progression, and the underlying pathological changes remain unknown.

Several clinical features strongly supported HPD rather than PsP in this case: (i) marked enlargement of the primary tumor, (ii) appearance of new liver metastases and pelvic lymphadenopathy, (iii) lack of tumor marker elevation (CEA and CA19-9), and (iv) rapid clinical decline, including worsening PS from 0 to 2, abdominal pain, and fever. Continuing ICI therapy to evaluate for PsP was therefore not a feasible option. Ultimately, switching to FOLFOXIRI plus bevacizumab led to significant tumor regression and clinical stabilization, demonstrating the benefit of timely therapeutic modification.

Emerging diagnostic tools, such as circulating tumor DNA analysis and immune profiling, may further improve the ability to differentiate HPD from PsP.^13,14)^ Early recognition of HPD and prompt treatment adaptation are essential to improve outcomes in patients receiving ICIs. Further studies are warranted to establish robust diagnostic criteria and predictive biomarkers, particularly for MSI-H and Lynch syndrome–associated cancers.

## CONCLUSIONS

We describe a rare case of HPD in a patient with Lynch syndrome receiving pembrolizumab. This case underscores the importance of early assessment of treatment response in MSI-H tumors and highlights the clinical need for reliable diagnostic criteria and predictive biomarkers to distinguish HPD from PsP. Continued research is essential to clarify the biological mechanisms underlying atypical responses to ICIs in Lynch syndrome–associated cancers and to optimize management strategies for this patient population.

## DECLARATIONS

### Availability of data and materials

The datasets supporting this case report are available from the corresponding author upon reasonable request.
